# Changes in T cell effector functions over an 8-year period with TNF antagonists in patients with chronic inflammatory rheumatic diseases

**DOI:** 10.1038/s41598-018-26097-x

**Published:** 2018-05-18

**Authors:** Ilaria Sauzullo, Rossana Scrivo, Paola Sessa, Fabio Mengoni, Vincenzo Vullo, Guido Valesini, Claudio Maria Mastroianni

**Affiliations:** 1 Department of Public Health and Infectious Diseases, ‘Sapienza’ University, Rome, Italy; 2 Department of Internal Medicine and Medical Specialties, Rheumatology Unit, ‘Sapienza’ University, Rome, Italy

## Abstract

The aim of the study was to clarify the effect of long-term anti-TNF therapy on T cell function in patients with rheumatologic immune-mediated inflammatory diseases (IMID). The production of IFNγ by T cells was evaluated at baseline and after 1, 2, 4, and 8 years of anti-TNF agents by means of a QuantiFERON-TB Gold In-Tube assay. The T cell proliferation and surface co-expression of CD25/CD134 in response to phytohaemagglutinin together with the *in vitro* impact of anti-TNF therapy on the functional capacity of T cells were evaluated after 8 years from the onset of the biological treatment. Age-matched healthy donors were enrolled as controls. The quantitative mitogen-induced IFNγ responses significantly increased with respect to baseline at each time point, apart from the determination after 4 years. We found an increased expression of CD25/CD134 in CD4^+^ compared to CD8^+^ T cells both in patients and controls. The *in vitro* addition of anti-TNF agents induced a significant decrease of both the IFNγ response and of CD25/CD134, whereas no effect on the intensity of the proliferative response was observed. Our data provide a biological basis for the reassuring issues on the safety of long-term anti-TNF treatment in patients with IMID.

## Introduction

Tumor necrosis factor (TNF) drives the early cytokine cascade at sites of inflammation^1^. TNF-targeted biological therapy with monoclonal antibodies (infliximab, adalimumab, golimumab, certolizumab pegol) or soluble receptors (etanercept) dramatically changed the course of several chronic inflammatory diseases such as rheumatoid arthritis (RA), psoriatic arthritis (PsA), ankylosing spondylitis, psoriasis (PsO), and inflammatory bowel disease (IBD)^2^. Most of the favorable outcomes of these agents have been attributed to their ability to antagonize the effects of TNF at late steps of the inflammatory cascade^3^.

TNF antagonists have been assessed for immune system effects, including *ex vivo* assays of cells from treated patients. Previous studies reported that these drugs suppress cytokine production by circulating effector T cells^4–11^, although an enhanced synthesis of TNF and interferon γ (IFNγ) by T cells upon *in vitro* stimulation has also been reported^12^. Likewise, Bosè *et al*. evidenced a dual effect in patients with PsO and IBD, in which TNF blockade was capable of inducing an augmented responsiveness of peripheral T cells to TCR-stimulation and an increased secretion of cytokines *ex vivo*, whereas in biopsies from target tissues TNF blockade led to a dramatic reduction of Th17 and Th1 responses^13^.

IFNγ release assays (IGRAs) provide the most recent method to diagnose latent tuberculosis (LTBI) infection and are increasingly used as a screening tool in patients undergoing treatment with anti-TNF agents^14^. IGRAs measure the cell-mediated immune response against some *M*. *tuberculosis* specific antigens and incorporate an internal positive control (phytohaemagglutinin, PHA), which tests the ability of T cells to produce IFNγ^15^. A recent meta-analysis showed that glucocorticoids, oral immunosuppressants and biological therapy produce more negative, but not indeterminate, IGRA results^16^. However, only five studies on anti-TNF agents were included in this meta-analysis^17–21^ and, among these, only two were performed in patients with autoimmune diseases, in which a clinical evaluation was done without a determination of the biological effects of anti-TNF agents on IGRA outcome^18,19^.

Therefore, we decided to clarify the *ex vivo* effect of long-term anti-TNF therapy on T cell effector function in subjects with rheumatologic immune-mediated inflammatory diseases (IMID). To this purpose, we performed multiple investigations comprising IFNγ secretion by means of an IGRA assay, and T cell proliferation and surface co-expression of CD25 and CD134 in response to PHA. Furthermore, we examined the *in vitro* impact of anti-TNF biological therapy with etanercept (Eta) or adalimumab (Ada) on the functional capacity of T cells.

## Methods

### Study population

Starting from 2008, we conducted a longitudinal, prospective, observational study on patients with IMID referring to the rheumatology outpatient clinic at Sapienza University of Rome, Italy, and candidates to an anti-TNF agent as their first biological treatment. Before starting anti-TNF agents, patients underwent screening for LTBI, including a postero-anterior chest radiograph and the QuantiFERON-TB Gold In-Tube (QFT-GIT) assay (Cellestis GmbH, QIAGEN Inc. Valencia, CA, USA), one of the currently available IGRAs. The original population consisted of 102 patients who were initially studied to assess possible QFT-GIT conversions and reversions in relation to the clinical outcome during the initial 18 months of treatment with biological therapy^22^. Thirty-three of these patients were also involved in another study with the aim to analyse CD4^+^ T cells by multi-functional flow cytometry during a total of 36-month follow-up of their treatment with biological therapy^23^. Among patients of this last group, only those continuing biological treatment for at least 8 years were included in the current study. At established time points, taken before (T0) and after 1 (T1), 2 (T2), 4 (T4), and 8 (T8) years since the onset of anti-TNF agents, blood samples were collected from IMID subjects to measure the IFNγ secretion. Blood samples obtained after 8 years of biological therapy were used to perform the proliferation assay, together with the co-expression of CD25/CD134 assay. These two tests, as well as the IFNγ secretion, were also performed in a group of healthy donors (HD). At the same time as the blood draws, in patients with IMID disease activity was calculated by using a validated index, the modified disease activity score (DAS28)^24^.

The study received approval from the Policlinico Umberto I Ethics Committee (reference number 2669). The study was conducted in accordance with approved guidelines and regulations, and all of the participants provided written informed consent.

### ***Ex vivo*** assays

#### QuantiFERON TB Gold-In Tube assay

Whole blood from IMID subjects was tested using the QFT-GIT assay (Cellestis GmbH, QIAGEN Inc. Valencia, CA, USA) according to the manufacturer’s instructions. The result was considered indeterminate if IFN γ level of mitogen minus that of the negative control was <0.5 IU/mL and/or if IFNγ level of the negative control was >8.0 IU/mL. Analysis of data was done with the QFT-GIT analysis software.

#### CD25/CD134 assay and flow cytometry procedures

Heparinized peripheral blood (0.5 ml) was mixed with an equal volume of RPMI-1640 Medium (Sigma-Aldrich, Germany), and then incubated in sterile 24-well culture plates with PHA (5 μg/mL, Sigma-Aldrich, Germany) or medium alone at 37 °C in 5% CO_2_ for 44–48 hours before antibody staining and analysis by flow cytometry.

After 44 hours of incubation, 100 μl of whole blood was stained with anti-CD3 PerCP, anti-CD4 PE-Vio770, anti-CD25 APC, anti-CD8 FITC (BD Biosciences, San Josè, USA), anti-CD134 PE (BioLegend, San Diego, CA, USA) for 20 min at 4 °C, followed by treatment with FACS lysing solution (BD Biosciences, according to manufacturer’s instructions) and washed twice with FACS buffer (PBS/0.5% BSA, 0.01% sodium azide). Cells were fixed in 1% paraformaldehyde and acquired within 1 hour using a MACSQuant Analyzer flow cytometer (MiltenyiBiotec, Germany) after calibration and automatic compensation. From each sample, a minimum of 100.000 events was collected and analyzed using FlowJo software v. 10 (Tree Star, San Carlos, CA, USA). Lymphocytes were identified on the basis of forward and side-scatter; after gating on CD3^+^CD4^+^ T cells and CD3^+^CD8^+^ T cells, an analysis of CD25 APC and CD134 PE staining events was performed. Standard gating procedures using fluorescence minus one (FMO) controls (unstimulated) were used to identify positive cells. Background CD25^+^/CD134^+^ expression in negative control was subtracted from stimulated condition. The cut-off for a positive response was calculated as the mean response (%) + 3 standard deviation (SD) above negative control from HD.

#### T cell proliferation assay and flow cytometry procedures with data analysis

Peripheral blood mononuclear cells (PBMCs) were isolated from whole blood of patients with IMID and HD by density-gradient centrifugation on Ficoll-Histopaque (Sigma-Aldrich, Germany). Fresh PBMCs, at a density of 1 × 10^6^ cells/mL, were labelled with 5 μM CellTrace Violet (CTV) fluorescent dye (Molecular Probes, Invitrogen, CA, USA) with immediate vortexing to ensure rapid and homogeneous labelling of cells. Cells were then incubated at 37 °C for 20 min and washed 3 times with RPMI-1640 Medium (Sigma-Aldrich, Germany) supplemented with 10% heat-inactivated fetal calf serum (FCS, Gibco, CA, USA). Fluorescent dye-labelled PBMCs were cultured at a concentration of 1.5 × 10^6^ cells/mL in RPMI-1640 Medium, supplemented with 2 mM L-glutamine, 1% of penicillin/streptomycin, 10% FCS, in the presence of PHA (5 μg/mL, Sigma-Aldrich, Germany) or medium alone at 37 °C in 5% CO_2_ for 5 days.

On day 5 of culture, the cells were stained with anti-CD3 PerCP, anti-CD4 PE-Vio770, anti-CD8 PE (MiltenyiBiotec, Germany) for 20 min at 4 °C, and fixed with 1% paraformaldehyde.

Samples were acquired within 1 hr using MACSQuant Analyzer Flow Cytometry (MiltenyiBiotec, Germany), after calibration and automatic compensation, and analyzed with Flow Jo software v. 10 (Tree Star, San Carlos, CA, USA). The proliferation fraction (PF) of gated CD4^+^ and CD8^+^ T cells was quantified based on CTV dilution and shown as percentage of CD4^+^ and CD8^+^ T cells in CTV^low^ gate. The gating strategy used to identify T cell proliferation is shown in Supplementary Fig. S1. The ΔPF was calculated subtracting the negative control PF from the PHA-induced PF. As negative controls, we used labelled unstimulated cells which provide a fluorescence measurement of non-proliferating lymphocytes. To normalize the values, we calculated the stimulation index (SI), which represents the proportion of cells proliferating in response to PHA divided by the proportion of cells proliferating spontaneously in medium. SI values > 2 were considered as positive proliferative response^25^.

### *In vitro* assay

For *in vitro* experiments, whole blood and fresh fluorescent dye-labelled PBMCs of 3 HD were cultured, as described above, in the presence of PHA (5 μg/mL). TNF blockers were added at trough and peak drug concentrations, and also at supratherapeutic concentration (Eta at 1, 2, and 5 μg/mL; Ada at 5, 10, and 15 μg/mL) reached in blood during therapy, on the basis of information provided by manufacturers^26,27^. Positive control wells contained PHA only, and negative control wells contained medium alone. All conditions were set up in triplicate wells.

After 18–24 hours of culture at 37 °C in 5% CO_2_, the supernatants were collected and IFNγ levels were measured according to the manufacturer’s instructions (Cellestis GmbH, QIAGEN Inc. Valencia, CA, USA). All data were background-corrected by subtracting measured IFNγ concentration of the negative sample from those of the PHA-stimulated sample. After 44 hours of incubation at 37 °C in 5% CO_2_, the frequency of CD25^+^CD134^+^ T cells was assessed as described above, in the presence or absence of the biological drug. After 5 days of culture at 37 °C in 5% CO_2_, T cell proliferation was assessed, as described above, in the presence or absence of the biological drug.

### Statistical analysis

Median (interquartile range, IQR) or mean (±SD) of the different parameters were calculated. Longitudinal analysis was evaluated with the non-parametric Wilcoxon-signed-rank test. Non-parametric Mann-Whitney test was used to compare the continuous demographic characteristics of IMID patients and HD and the T cell responses between two groups. Pearson correlation coefficient was used to examine the correlation between IFNγ secretion assay and parameters from flow cytometric analysis (T cell proliferation and co-expression of CD25^+^/CD134^+^). Student’s *t* test was used for statistical analysis of data from *in vitro* experiments with TNF antagonists. All statistical analyses were two-sided, performed using GraphPad Prism Software v. 5 (Software MacKiev), and considered significant at *p*-values < 0.05.

### Data Availability

The datasets generated and/or analysed during the current study are available from the corresponding author on reasonable request.

## Results

### Study subjects

The main characteristics of the patients included in the study and their therapeutic profile are summarized in Table 1. Of the 33 patients participating in the multi-functional flow cytometry study^23^, only 15 had a complete 8-year follow-up and were considered for the present study. The reasons for discontinuation were the following: withdrawal of biological treatment due to inefficacy or adverse events (n = 15), refusal to allow a blood draw (n = 2), lost-to-follow-up (n = 1). Of the enrolled patients, 7 had a diagnosis of RA and 8 of PsA according to standard criteria^28,29^. Ten patients (66%) initiated treatment with Eta and 5 (33%) with Ada. Eleven age-matched HD were enrolled as controls (Table 1).

**Table 1:** Demographics and clinical characteristics of the patients after eight years of anti-TNF biological treatment.

	IMID	HD
	(n = 15)	(n = 11)
**M/F (n)**	7/8	6/5
**Age (years; median/IQR)**	60/52-68	51/41-65^*^
Underlying disease (n)		
Rheumatoid arthritis	7	—
Psoriatic arthritis	8	—
Concomitant treatment regimen (n/%)		
Biologics	6/40	—
Biologics + glucocorticoids	4/27	—
Biologics + DMARDs	4/27	—
Biologics + DMARDs + glucocorticoids	1/6	—

^*^Statistical analysis was performed using the Mann-Whitney test (p = 0.15).

Note: IMID = immune-mediated inflammatory diseases; HD = healthy donors; IQR = interquartile range; DMARDs = disease modifying anti-rheumatic drugs (include methotrexate and leflunomide).

### ***Ex vivo*** assays

#### Impact of biological agents on the mitogen-induced IFNγ response

In order to study the effect of long-term TNF antagonist treatment on T cell response, we first analyzed the ability of IMID subjects to secrete IFNγ by comparing both the rate of indeterminate QFT-GIT results and the quantitative mitogen-induced IFNγ responses, corresponding to the maximum IFNγ response, at different time points, i.e. before and after 1, 2, 4 and 8 years of biological therapy.

The prevalence of indeterminate QFT-GIT results did not significantly change in the cohort of patients over the long-term follow-up (p > 0.05), indicating that the performance of this assay was not affected by the biological therapies (Table 2).

**Table 2:** Changes occurred in the quantitative mitogen-induced IFNγ responses from QFT-GIT taken at the different time points during treatment with TNF antagonists.

	QFT-GIT (IU/mL) results during biological treatment				
	Baseline*	1 year	2 years	4 years	8 years
All patients (n = 15)	6.3 (2.7–16.9)	11.2 (8.09–14.6)	15.7 (10–30.4)	6.3 (0.98–15.8)	14.2 (13–16.9)
Indeterminate results (n)	0	0	1	2	0
Etanercept (n = 10)	7.4 (2.7–19.3)	11.03 (4.8–24.5)	15.1 (9.6–30.5)	2.07 (0.7–12.9)	13.8 (11.7–16.9)
Adalimumab (n = 5)	5.06 (2.6–8.9)	11.8 (8.6–14.3)	20.5 (8.5–31.3)	8.4 (5.2–16)	14.3 (9.9–18.5)
RA (n = 7)	6.3 (2.7–16.9)	11.2 (2.6–12.7)	12.2 (8.7–15.7)	1.4 (0.4–6.9)	14.2 (13–17.7)
PsA (n = 8)	5.7 (2.8–16.8)	11.5 (9.1–15.9)	29.5 (12.6–31)	13.9 (4.7–15.9)	14.02 (7.8–16.8)

^*^Prior to the onset of biological treatment. The values expressed as IU/ml are median with interquartile range.

Note: QFT-GIT = QuantiFERON-TB Gold In-Tube; RA = rheumatoid arthritis; PsA = psoriatic arthritis.

**Figure 1:**
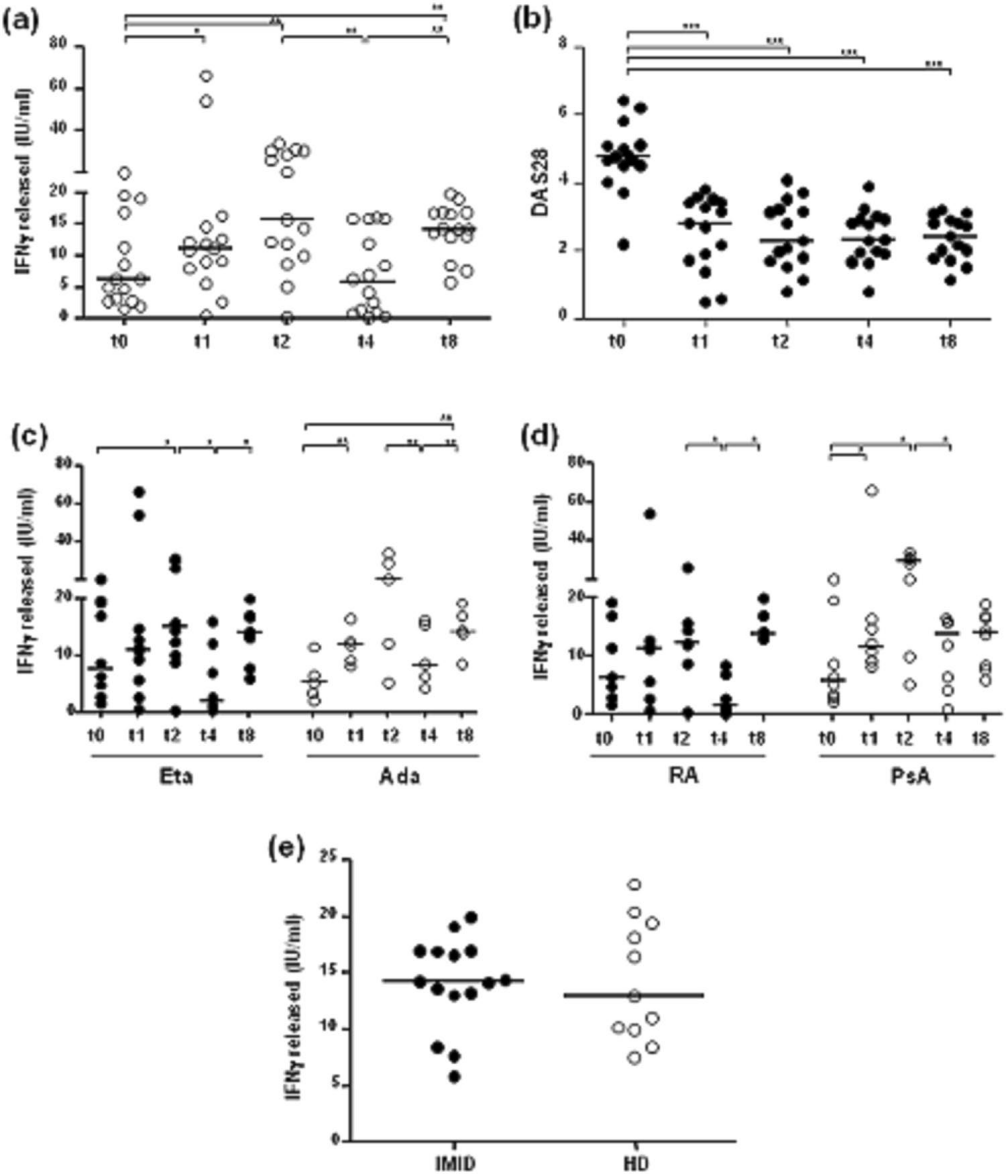
Mitogen-induced IFNγ response from patients undergoing treatment with TNF antagonists. The release of IFNγ in response to phytohemagglutinin (PHA) was evaluated after 18 hours of incubation using the QuantiFERON TB Gold-In Tube (QFT-GIT; Cellestis GmbH, QIAGEN Inc. Valencia, CA, USA) assay, according to the manufacturer’s instructions. The figure shows the mitogen-induced IFNγ response during follow-up. **(a)** IFNγ response (IU/mL) after mitogen stimulation was assessed in 15 IMID subjects before and after 1, 2, 4, and 8 years of biological agents. **(b)** Changes in the DAS28 score in the IMID subjects at the different time points of the follow-up period. **(c)** The analysis of mitogen-induced IFNγ response was assessed according to the type of biological agent in patients treated with Eta (n = 10, black circle) and in those treated with Ada (n = 5, white circle). **(d)** The analysis of mitogen-induced IFNγ response was assessed according to the type of inflammatory disease in 7 patients with rheumatoid arthritis (RA, black circle) and in 8 patients with psoriatic arthritis (PsA, white circle). **(e)** The IFNγ response was assessed by comparing IMID subjects (n = 15) after 8 years of treatment with TNF antagonists and healthy donors (HD, n = 11). Horizontal bars represent the median values. Statistical analysis was calculated by Wilcoxon Rank test for longitudinal data and by non-parametric Mann-Whitney test to compare IMID and HD subjects. Significant *p*-values are indicated.

Conversely, significant changes occurred in the quantitative mitogen-induced IFNγ responses following treatment with TNF antagonists. In particular, as shown in Fig. 1a, the IFNγ response in IMID patients increased significantly at T1 (p = 0.03) and T2 (p = 0.003) with respect to baseline, decreased significantly at T4 with respect to T2 (p = 0.002), and showed a further significant increase at T8 with respect to T4 (p = 0.0015) and to baseline (p = 0.03). The levels of IFNγ at T4 were similar to those observed at baseline. The mitogen-induced IFNγ response increased 0.77 and 1.49-fold at T1 and T2, respectively; significantly decreased by 60% (0.6-fold, from 15.77 to 6.34 IU/mL) at T4 and increased 1.25-fold at T8.

The DAS28 values decreased significantly, with respect to baseline (median 4.76, IQR 4.5–5.1), at T1 (median 2.79, IQR 1.7–3.4), T2 (median 2.27, IQR 1.6–3.2), T4 (median 2.31, IQR 1.9–2.9), and T8 (median 2.4, IQR 1.7–2.8) (p < 0.0001 for all comparisons) (Fig. 1b).

Next, the quantitative mitogen-induced IFNγ response was evaluated according to the type of biological agent or the inflammatory disease. The IFNγ response showed a similar trend irrespective of the treatment (Eta or Ada) and the disease (RA or PsA) (Fig. 1c,d) during the 8-year course of follow-up. In particular, we observed a decrease of mitogen-induced IFNγ response after 4 years of therapy, mainly in patients treated with Eta (p = 0.019) and in those with RA (p = 0.046) with respect to time point T2.

The IFNγ response was also analyzed by comparing all IMID subjects after 8 years of treatment with TNF antagonists and HD. The responses induced by PHA in patients (median 14.2 IU/mL, IQR 13–16.9) were in the same range as those of HD (median 13 IU/mL, IQR 10–19.3; p = 0.95) (Fig. 1e). Serial numerical changes occurred in the mitogen-induced IFNγ responses from QFT-GIT of IMID patients at the different time points during treatment with TNF antagonists are summarized in Table 2.

#### Impact of biological agents on the CD25/CD134 assay

**Figure 2:**
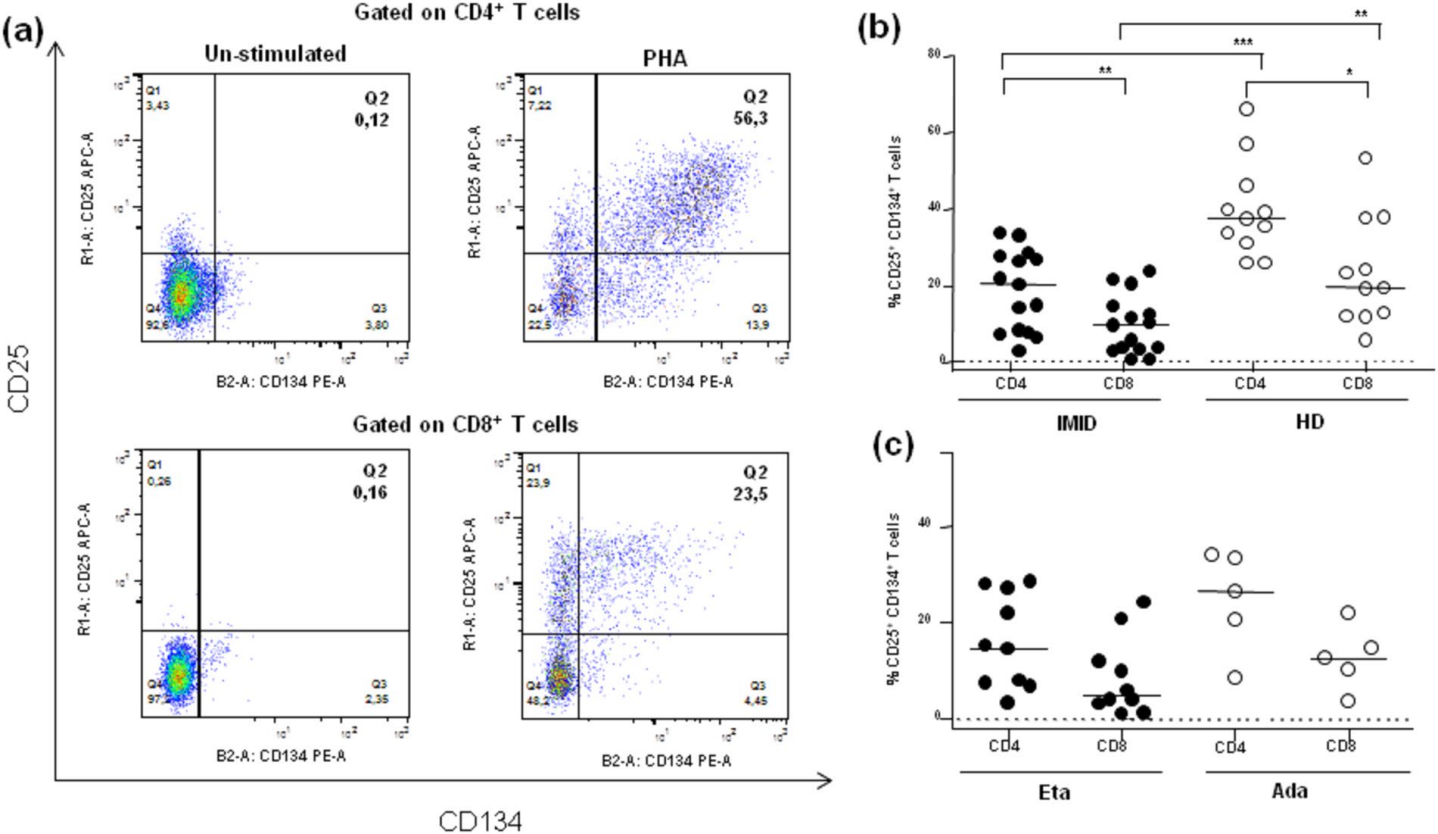
Characterization of the *ex vivo* CD25^+^CD134^+^ T cell assay. (**a**) Representative dot plots from healthy donors (HD) showing the staining for CD25^+^CD134^+^ T cells in whole blood cultures in response to either control (*left*) or phytohemagglutinin (PHA) at 5 μg *(right*). The percentages of CD25^+^CD134^+^ T cells are shown in upper right quadrants. **(b)** Frequencies of CD25^+^CD134^+^ T cells in response to PHA in 15 IMID patients after 8 years of treatment with TNF antagonists and in 11 HD are shown. Statistical analysis was performed using the Mann-Whitney test to compare IMID and HD subjects and significant *p*-values are indicated. **(c)** Frequencies of CD25^+^CD134^+^ T cells were assessed according to the type of biological agent. Statistical analysis was performed using the Mann-Whitney test to compare subjects treated with Eta and Ada. Horizontal bars represent the median values and horizontal dashed lines indicate the cut-off of positive response corresponding to 0.47% (mean 0.098, SD 0.12) for CD4^+^ T cells, and 0.41% (mean 0.11, SD 0.09) for CD8^+^ T cells. The cut-off for a positive response was calculated as the mean response (%) ± 3SD of negative control.

Co-expression of CD25 and CD134 by T cells in PHA-stimulated whole blood cultures of 44 hours duration was assessed in IMID patients after 8 years of biological therapy. Background co-expression of CD25 and CD134 on T cells was extremely low, as shown in a representative un-stimulated 44 hour-whole blood culture (Fig. 2a). Un-stimulated cultures from HD were used to determine the cut-off for a positive response, designed as the mean response (%) + 3 SD of the negative control.

Flow cytometric analysis in IMID patients showed frequencies of PHA-stimulated CD25^+^CD134^+^CD4^+^ T cells (median 20.4%, IQR 7.7–27.8%) higher than CD25^+^CD134^+^CD8^+^ T cells (median 9.82%, IQR 3.5–14.8%; p = 0.0181) (Fig. 2b). Indeed, based on positive cut-off, corresponding to 0.47% (mean 0.09, SD 0.12) for CD4^+^ T cells, and 0.41% (mean 0.11, SD 0.09) for CD8^+^ T cells, we scored as positive all 15 IMID patients assessing both the CD4^+^ and CD8^+^ T cells.

We also compared the co-expression of surface markers between patients treated with Eta and Ada. Again, the co-expression of CD25 and CD134 was mostly present on CD4^+^ than CD8^+^ T cells with either treatments (p < 0.05 for all comparisons). However, patients treated with Ada showed a trend towards more activated T cells (median 26.5%, IQR 14.4–33.6% for CD4^+^ and median 12.5%, IQR 6.9–18.3% for CD8^+^) as compared to those treated with Eta (median 14.6%, IQR 7–27.2% for CD4^+^ and median 4.8%, IQR 2.5–13.9% for CD8^+^) (p = 0.16 for CD4^+^ and p = 0.25 for CD8^+^; Fig. 2c).

Among HD, a high percentage of PHA-stimulated CD25^+^CD134^+^ in both T cell subsets was observed, with frequencies of CD4^+^ T cells (median 37.8%, IQR 31.6–46.5%) greater than those of CD8^+^ T cells (median 19.7%, IQR 12.1–38.1%; p = 0.01). Finally, the frequency of CD4^+^ and CD8^+^ T cells positive for CD25 and CD134 was significantly higher in HD as compared to IMID subjects (p = 0.0004 for CD4^+^ and p = 0.0059 for CD8^+^) (Fig. 2b).

No correlation was found between DAS28 and the frequency of CD25^+^CD134^+^CD4^+^ T cells (r = −0.19, p = 0.49, r^2^ = 0.03) or CD25^+^CD134^+^CD8^+^ T cells (r = 0.005, p = 0.98, r^2^ = 0) (data not shown).

#### Impact of biological agents on the T cell proliferation assay

**Figure 3:**
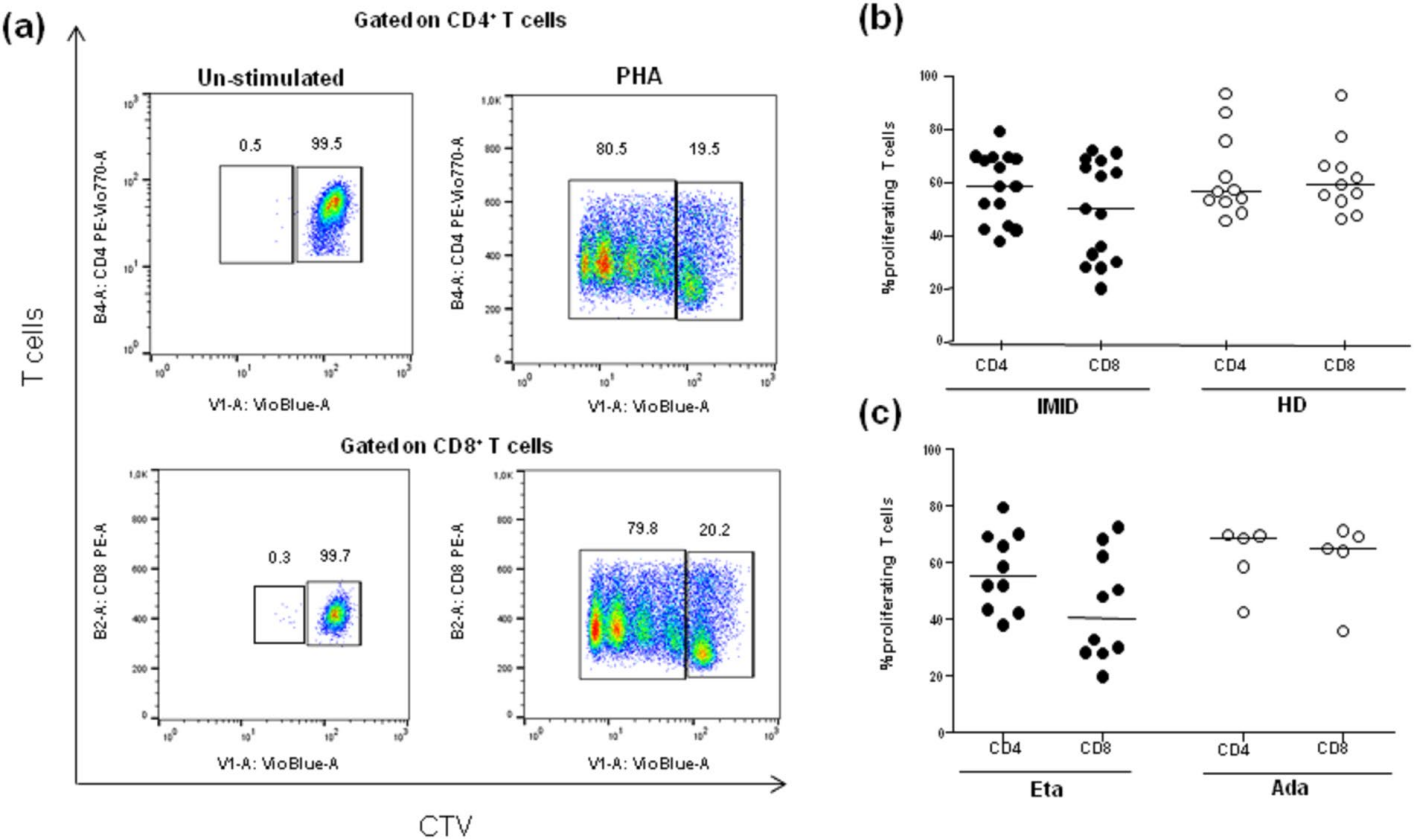
*Ex vivo* proliferation assay of CD4^+^ and CD8^+^ T cells. (**a**) Representative proliferation dot plots of CD4^+^ and CD8^+^ T cells in healthy donors (HD). The proliferation was assessed staining PBMCs with the fluorescent dye Cell Trace Violet (CTV) and determining the CTV content of CD4^+^ and CD8^+^ T cells after 5 days of stimulation with medium alone (*left*) or phytohemagglutinin (PHA) at 5 μg (*right*). In response to PHA, the figure depicts CD4^+^ and CD8^+^ T cells that have divided 3–4 times based on CTV dilution intensity; in contrast, in medium alone, the T cells did not show any divisions. **(b)** Cumulative analyses of the frequency of CD4^+^ and CD8^+^ T cells endowed with the proliferation ability in response to PHA in IMID patients (n = 15) and HD subjects (n = 11). Statistical analysis was performed using the Mann-Whitney test to compare IMID and HD subjects and significant *p*-values are indicated. **(c)** Frequencies of proliferating T cells were assessed according to the type of biological agent. Statistical analysis was performed using the Mann-Whitney test to compare subjects treated with Eta and Ada. The proliferation of T cells was expressed as the percentage of CD4^+^ and CD8^+^ T cells in CTV^low^ gate. Horizontal bars represent the median values.

The proliferative capacity of CD4^+^ and CD8^+^ T cells from IMID patients was measured after 8 years of biological treatment and data were compared to those obtained from HD. After inducing proliferation, T cells were identified by flow cytometry as a result of fluorescence diminution from CTV labelling. Figure 3a shows CTV-fluorescence obtained stimulating PBMCs with PHA for 5 days. Cells proliferating in response to PHA were clearly detectable, as their CTV-fluorescence intensity was lower than the major population in the parental generation, and was expressed as percentage of CD4^+^CTV^low^ and CD8^+^CTV^low^ T cells. Gating CD3^+^CD4^+^ and CD3^+^CD8^+^ T cells separately, we observed a positive proliferative response (SI > 2) to PHA, with frequencies ranging from 37.9% to 79.5% for CD4^+^ T cells, and from 20% to 72.4% for CD8^+^ T cells in IMID individuals. The comparison of proliferative responses between CD4^+^ and CD8^+^ T cells among treated subjects did not reveal any significant differences (median 58.7% *vs* 50.43%, p = 0.17) (Fig. 3b).

When comparing patients treated with Eta and Ada, a constantly high proliferative capacity in both T cell subsets was observed. Interestingly, CD4^+^ and CD8^+^ T cells in Ada subjects were characterized by greater proliferative responses (median 68.6% for CD4^+^, 65.04% for CD8^+^) than those observed in patients treated with Eta (median 55.3% for CD4^+^, 40.5% for CD8^+^), even if the difference was not statistically significant (p = 0.59 for CD4^+^ and p = 0.09 for CD8^+^) (Fig. 3c).

Next, we assessed the proliferative capacity of T cells among HD. The proliferative frequencies were similar, ranging from 47% to 93% for CD4^+^ T cells, and from 46.8% to 93.1% for CD8^+^ T cells. No significant differences were observed between CD4^+^ and CD8^+^ T cells (median 57.8% *vs* 59.8%, p = 0.94) (Fig. 3b). The comparison of proliferative CD4^+^ and CD8^+^ T-cell responses between IMID and HD did not reveal any significant differences (median 58.7% *vs* 57.8% p = 0.71 for CD4^+^; median 50.43% *vs* 59.8%, p = 0.23 for CD8^+^) (Fig. 3b).

#### Correlations among IFNγ secretion, expression of CD25/CD134, and T cell proliferation

**Figure 4:**
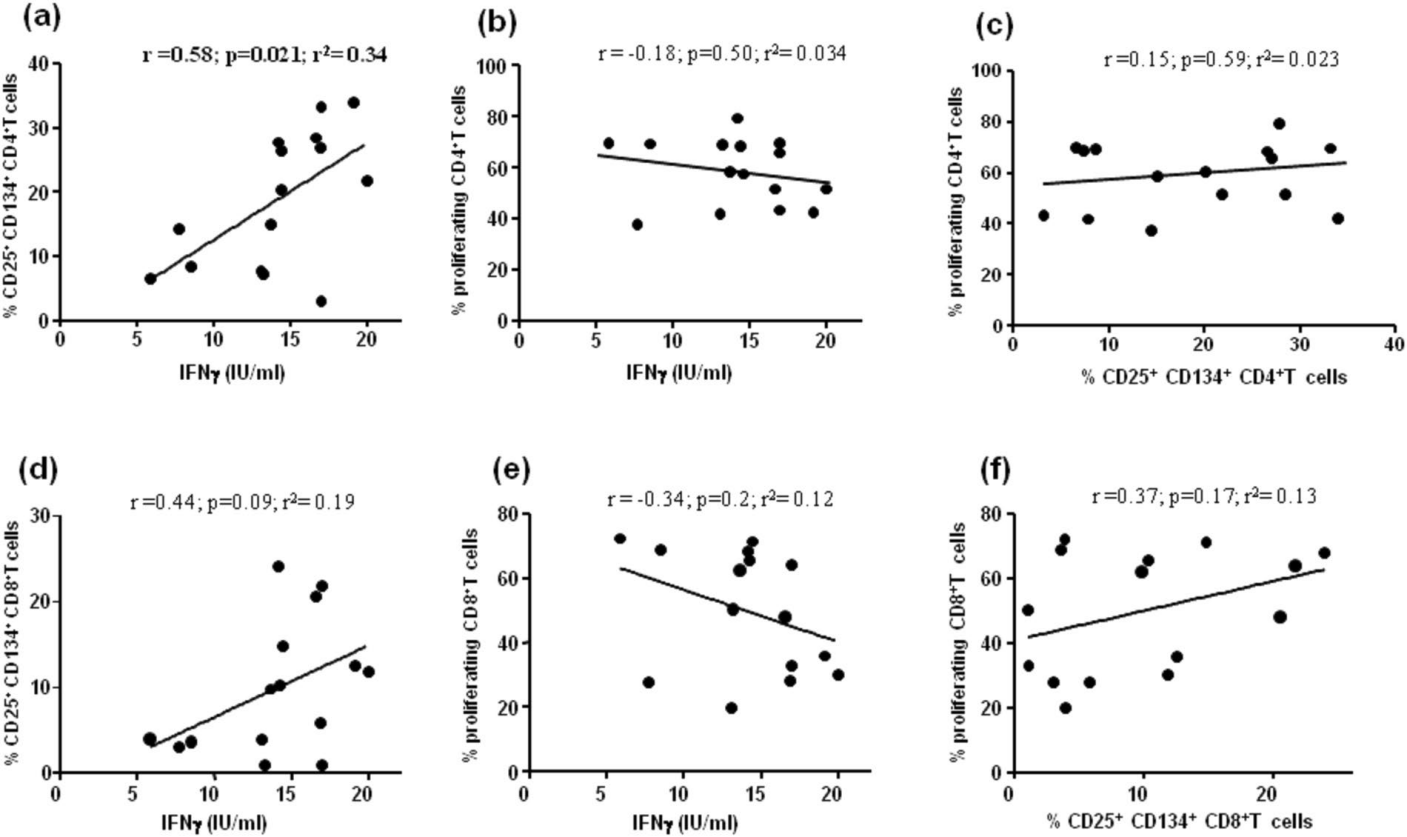
Correlations between IFNγ secretion, co-expression of activation markers, and lymphocyte proliferation in response to phytohemagglutinin. The *ex vivo* immunologic assays of all IMID subjects were correlated using Pearson correlation coefficients. **(a)** IFNγ response to phytohemagglutinin (PHA) was positively correlated with the frequency of CD25^+^CD134^+^ CD4^+^ T cells (r = 0.58, p = 0.028, r^2^ = 0.34), **(b)** but not with the CD4^+^ T cells proliferating in response to PHA (r = −0.18, p = 0.5, r^2^ = 0.034). (**c)** Lack of correlation between frequencies of CD25^+^CD134^+^ CD4^+^ T cells and CD4^+^ T cell proliferation (r = 0.15, p = 0.5, r^2^ = 0.023). (**d–f)** Lack of correlation in all CD8^+^ T cell analysis.

We examined possible correlations among cytokine production, activation and proliferation of T cells in IMID patients (Fig. 4). IFNγ response was positively correlated with the frequency of CD25^+^CD134^+^CD4^+^ T cells (r = 0.58, p = 0.021, r^2^ = 0.34) (Fig. 4a), but not with the proliferating CD4^+^ T cells in response to PHA (r = −0.18, p = 0.5, r^2^ = 0.034) (Fig. 4b). In addition, no correlation was found between the frequencies of CD25^+^CD134^+^CD4^+^ T cells and CD4^+^ T cell proliferation (r = 0.15, p = 0.5, r^2^ = 0.023) (Fig. 4c). The correlations for CD8^+^ T cell analysis were not significant (Fig. 4d–f). No significant correlation was observed in HD, except for the one between the IFNγ response and CD25^+^CD134^+^CD8^+^ T cells (r = 0.63, p = 0.035, r^2^ = 0.40) (data not shown).

### ***In vitro*assays**

In another set of experiments, we determined whether the functional capacity of T cells could be affected by the addition of anti-TNF agents in culture. PBMCs and whole blood of 3 HD were stimulated with PHA in the absence or presence of increasing concentrations of TNF antagonists to examine the IFNγ response, the surface co-expression of CD25 and CD134, and the proliferative capacity of T cells (Table 3).

**Table 3:** Results from *in vitro* experiments: evaluation of IFNγ response, co-expression of CD25/CD134, and proliferation of T cells.

	*In vitro* effects of biological therapy				
	IFNγ (IU/mL)	% CD25^+^CD134^+^ T cells		% proliferating T cells	
		CD4^+^	CD8^+^	CD4^+^	CD8^+^
No treatment	19.3 ± 2.5	58.9 ± 20.9	41.6 ± 17.6	66.1 ± 16.8	70.8 ± 10.1
Eta (1 μg/mL)	19 ± 2.9	43.2 ± 27.5	24.0 ± 16.8	71.4 ± 17.5	71.5 ± 6.3
Eta (2 μg/mL)	18.5 ± 1.6	41.0 ± 26.9	23.7 ± 17.5	71.2 ± 12.8	73 ± 6.5
Eta (5 μg/mL)	14.6 ± 5.1	48.5 ± 17.8	25.2 ± 12.8	70.2 ± 21.6	81.8 ± 14
No treatment	20 ± 0.2	37.1 ± 1.1	12.3 ± 0.5	88 ± 5.1	86.4 ± 11.4
Ada (5 μg/mL)	15 ± 2.9	21.9 ± 5.2	9 ± 1.9	86.9 ± 4.2	84.9 ± 7.7
Ada (10 μg/mL)	11.8 ± 3.3	23.2 ± 4.5	7.3 ± 1.6	85.9 ± 5.1	83.1 ± 9
Ada (15 μg/mL)	12.4 ± 2.5	18.5 ± 0.8	6.1 ± 1	86.1 ± 4.2	82.2 ± 11.5

The results are expressed as mean ± standard deviation. Note: Eta = etanercept; Ada = adalimumab.

The addition of different concentrations of Eta resulted in no effects on IFNγ levels compared with PHA-stimulated culture as positive control (p = 0.2) (Supplementary Fig. S2). In contrast, a significant decrease in the IFNγ release was observed in the presence of all concentrations of Ada, with the greatest effect seen at peak (10 μg/mL) and supratherapeutic concentration of the drug (15 μg/mL) (p = 0.0032) (Supplementary Fig. S2).

We further examined the co-expression of CD25 and CD134 in lymphocyte subsets upon addition of increasing drug concentrations after 44 hours of whole blood culture. A slight, albeit not significant, decrease of the co-expression of CD25 and CD134 on both T cell subsets was observed in the presence of all Eta concentrations (p = 0.7 for CD4^+^ and p = 0.3 for CD8^+^) (Supplementary Fig. S2).

Conversely, in the presence of Ada, the expression of surface markers was reduced to approximately half of the control values on both T cell subsets (p = 0.001 for CD4^+^ and p = 0.003 for CD8^+^) (Supplementary Fig. S2). Interestingly, the decrease was observed at both trough and at peak drug concentrations of Ada, but the greatest effect was seen at supratherapeutic concentration (15 μg/mL), in which the frequency of CD25 and CD134 decreased from 37.2% to 18.5% among CD4^+^ T cells (p = 0.001) and from 12.3% to 6.1% among CD8^+^ T cells (p = 0.003). Thus, these data show that Ada decreased the *in vitro* response in a dose-dependent fashion.

Finally, we investigated the *in vitro* influence of anti-TNF agents on the proliferation of PHA-stimulated lymphocytes after 5 days of incubation. The cell viability, assessed by trypan blue (Sigma-Aldrich, Germany), was >95% for cells after 44 hours of incubations, and decreased to 75% after 5 days in all culture conditions, irrespective of the anti-TNF agent used. The addition of TNF antagonists revealed no significant changes in the proliferative response of CD4^+^ and CD8^+^ T cells with respect to PHA-stimulated controls, showing a similar trend with Eta and Ada (p > 0.05 for all comparisons) (Supplementary Fig. S2).

In summary, the addition of TNF antagonists *in vitro*, especially Ada, resulted in a decrease of IFNγ response, paralleled by the decrease of the co-expression of CD25 and CD134 on T cell subsets, whereas no effect on the intensity of the proliferative response, regardless of the drug used, was observed (p > 0.05 for all comparisons).

## Discussion

This is the first long-term study examining the *ex vivo* and *in vitro* effects of TNF antagonists on T cell activation and function in IMID patients over an 8-year treatment period. We previously observed dynamic changes in IFNγ levels from QFT-GIT test performed in patients with rheumatic diseases during the initial 18 months of treatment with biological therapy^22^. This observation, not associated with modifications in clinical status of the patients, made the input to prolong the follow-up in order to investigate whether a longer treatment with TNF antagonists in patients with IMID might affect the function of T cells and favor the emergence of adverse clinical consequences. We restricted our observation to patients treated for at least 8 years. First of all, we aimed at evaluating the effector function of T cells at established time points (at baseline, and after 1, 2, 4, and 8 years of treatment with anti-TNF agents) by means of QFT-GIT. Interestingly, while no differences were observed in the rate of indeterminate results, the quantitative mitogen-induced IFNγ responses significantly increased with respect to baseline at each time point except for T4 determination, when IFNγ levels were comparable to baseline. However, neither clinical events were observed at any time points throughout the study nor technical reasons may be advocated to explain the mitogen impairment at T4. Because these results were confirmed irrespective of the treatment (Eta or Ada) and disease (RA or PsA), it seems that TNF antagonists do not impair the effector function of T cells, although the source of IFNγ production (i.e. NK cells) could not be precisely defined by using a whole blood assay^30^. These data are in agreement with those reported in several studies in patients with RA or spondyloarthritis^12,13,31–33^ in which, however, the follow-up ranged from 28 days to 6 months, being indeed far shorter than ours. The increased IFNγ expression upon long-term anti-TNF treatment probably finds a biological basis on the observation that chronic TNF exposure makes Th1 cells hyporesponsive, due to an attenuated calcium mobilization after T cell receptor activation; consequently, blocking endogenous TNF might have the effect of enhancing T cell responsiveness, thus reversing the T cell anergy induced by TNF exposure^31,34^. Consistent with this hypothesis, we subsequently found an increased expression of surface activation markers on both CD4^+^ and CD8^+^ T cells, as well as a valuable level of proliferating T cells. In particular, the stimulation of T cells by antigen or mitogen resulted in the up-regulation of CD25 (interleukin-2 receptor alpha, IL-2Rα) and CD134 (a TNF receptor superfamily member) surface activation markers, as measured by a flow cytometry-based assay^35^. We decided to evaluate CD25 and CD134 expression since these markers were recognized to be extremely sensitive and specific tools for the assessment of functional response by individual T cell subsets to a variety of stimuli^35^. Indeed, interactions between CD134 and its ligand are critical for memory T cell development and effector T cell survival^36^, and the up-regulation of the high-affinity IL-2Rα via CD25 is critical for the proliferation and differentiation of T cells^37^. In the current study, we used the CD25/CD134 assay to measure a broad polyclonal response to PHA mitogen, providing a simple assay of T lymphocyte function. We found a relatively high proportion of activated T cells in response to PHA in IMID patients, with a slightly enhanced expression of CD25 and CD134 markers on T lymphocytes upon Ada therapy. These findings parallel those of a recent report showing a significant increase in the expression of activation markers during the course of anti-TNF treatment^13^. In addition, among our IMID subjects, the co-expression of activation markers showed a positive correlation with IFNγ production. Conversely, no correlation between the co-expression of activation markers and T cell proliferation was found, in line with the observation of similar levels of proliferating T cells between IMID subjects and HD. Indeed, only a small increase in CD4^+^ and CD8^+^ T cell proliferation rate was observed following Ada treatment, as previously reported^31^, although in other studies circulating CD4^+^ T lymphocyte numbers remained unaffected in patients treated with TNF antagonists^38,39^, including Ada^40^.

While our *ex vivo* findings are compatible with the assumption that TNF is a negative regulator of T cell function^13^, we achieved opposite results when evaluating the *in vitro* impact of anti-TNF agents. In fact, the addition of these drugs, especially Ada, induced a significant decrease of both the IFNγ response and the co-expression of CD25 and CD134 on CD4^+^ and CD8^+^ T cells, whereas no effect on the intensity of the proliferative response, regardless of the drug used, was observed. This finding cannot be explained by a possible toxic effect of Ada, since the cell death rate was less than 25% after 5 days of stimulation in all culture conditions. Indeed, although there was a slight decrease in vitality, no difference of cell viability between the two different conditions was observed. This finding was confirmed by the observation that the proliferative response of CD4^+^ and CD8^+^ T cells, with respect to PHA-stimulated controls, did not reveal any significant differences between Ada and Eta. Interestingly, conflicting effects of T cells by different anti-TNF molecules have been described in other studies showing that the monoclonal anti-TNF antibodies infliximab and Ada inhibited *in vitro* the T cell activation and IFNγ production, whereas the soluble TNF receptor Eta did not^9,11^. In this respect, measuring serum drug levels at the different time points could have been of help in interpreting our results; however, this evaluation was not performed.

The stability of immune function observed over time in our patients receiving Eta is in agreement with the results of a study showing no significant differences between patients treated with Eta or placebo in the surface antigen phenotypes of peripheral blood leukocytes, T cell proliferative responses, neutrophil function, delayed-type hypersensitivity reactions, serum immunoglobulin levels, or incidence of infections^41^.

The different effects of Ada and Eta on the biological functions of T cells may indicate that, despite sharing a common therapeutic target, the anti-TNF agents may have different mechanisms of action depending on their molecular structure, while the differences between the *ex vivo* and *in vitro* outcome could reflect the distinct microenvironment supporting the biological response.

Overall, our data, though related to a small cohort of patients and the absence of a group of IMID patients not treated with anti-TNF treatment, do not show an impairment in the lymphocyte function over the long-term treatment with anti-TNF agents with respect to baseline, prior to the onset of biological agents. Furthermore, the small sample size did not allow evaluating the different effects on T cells of the combination therapy with glucocorticoids and/or conventional immunomodulators. In line with this observation, we did not observe any adverse effects during the course of treatment and, accordingly, recent studies at low risk of bias did not find an increased risk of infections in patients with RA treated with TNF antagonists^42,43^. Furthermore, the immune response following vaccination was comparable between IMID patients treated with TNF blockers and healthy controls, and formal guidelines for the use of vaccines in patients with IMID have been published^44^.

In conclusion, our study indicates that the long-term treatment with anti-TNF agents does not affect the functional capacity of T cells; rather, it seems that TNF antagonists may favor the restoration of the cellular immune function as evidenced by the increase of IFNγ production, the presence of activated T cells and the good proliferative capacity of T cells compared to HD. These results provide the biological basis for the reassuring issues on the safety of long-term anti-TNF treatment in patients with IMID.

## Electronic supplementary material

Supplementary Figure S1.

Supplementary Figure S2.
