# Supplementary material for: Changes in T cell effector functions over an 8-year period with TNF antagonists in patients with chronic inflammatory rheumatic diseases
Source: Sci Rep. 2018 May 18;8:7881. doi: 10.1038/s41598-018-26097-x (PMC5959893; doi:10.1038/s41598-018-26097-x)
Supplement: Supplementary file 1 — Supplementary Figure S1. [file 41598_2018_26097_MOESM1_ESM.pdf]

Supplementary information (S1)

Title: Changes in T cell effector functions over an 8-year period with TNF antagonists in patients with chronic inflammatory rheumatic diseases

Authors: Ilaria Sauzullo*[1], Rossana Scrivo[2], Paola Sessa[2], Fabio Mengoni[1], Vincenzo Vullo[1], Guido Valesini[2], Claudio Maria Mastroianni[1]

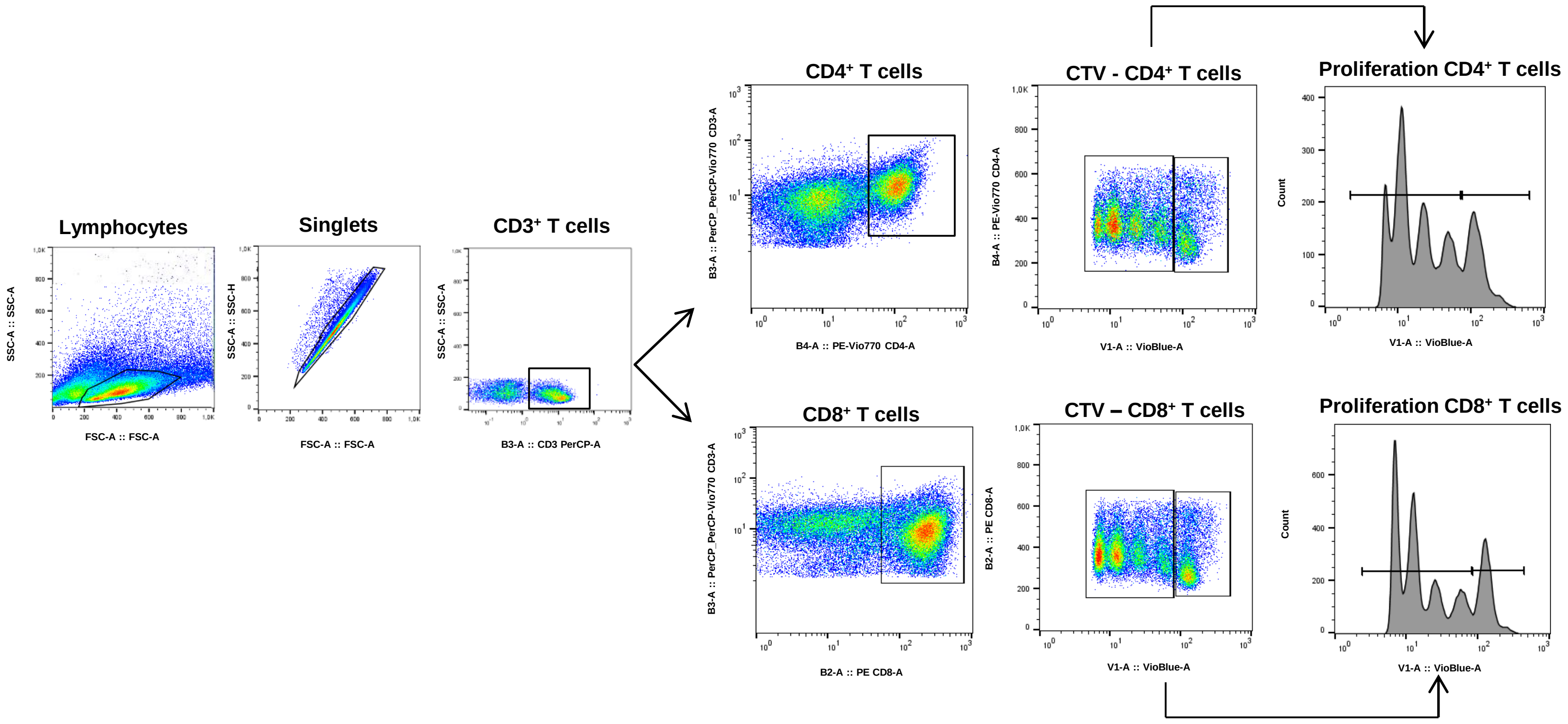

Supplementary Figure S1. Representative flow cytometry analysis showing the gating strategy for the analyses of T cell proliferation.

Lymphocytes were gated according to the forward (FSC) and side scatter (SSC) signals of the lymphocyte population (to exclude debris but include lymphocytes as they increase in scatter intensity upon proliferation). Cells were then gated on CD3 and the fluorescence intensity for both $CD4^{+}$ and $CD8^{+}$ was measured. The events within the gates were analyzed for Cell Trace Violet (CTV) and were plotted in the histograms. Proliferating cells are CTV^{low}. Histograms illustrate CTV $CD4^{low}$ and $CD8^{low}$ T cell proliferation in response to phytohemagglutinin (PHA).
