# Supplementary material for: Changes in T cell effector functions over an 8-year period with TNF antagonists in patients with chronic inflammatory rheumatic diseases
Source: Sci Rep. 2018 May 18;8:7881. doi: 10.1038/s41598-018-26097-x (PMC5959893; doi:10.1038/s41598-018-26097-x)
Supplement: Supplementary file 2 — Supplementary Figure S2. [file 41598_2018_26097_MOESM2_ESM.pdf]

Supplementary information (S2)

Title: Changes in T cell effector functions over an 8-year period with TNF antagonists in patients with chronic inflammatory rheumatic diseases

Authors: Ilaria Sauzullo*[1], Rossana Scrivo[2], Paola Sessa[2], Fabio Mengoni[1], Vincenzo Vullo[1], Guido Valesini[2], Claudio Maria Mastroianni[1]

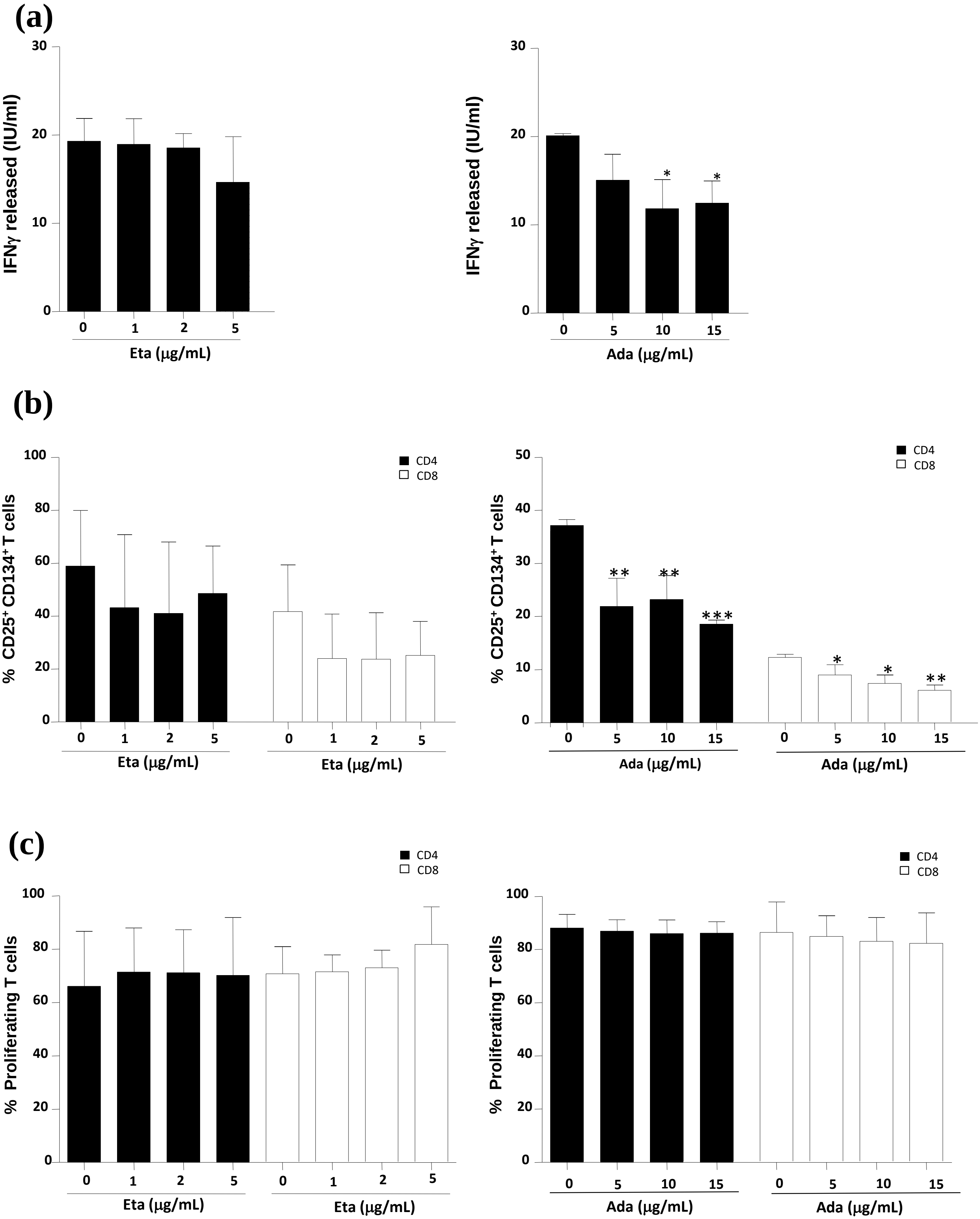
(a)
IFNγ released (IU/ml)
30
20
10
0
0
1
2
5
Eta (μg/mL)
IFNγ released (IU/ml)
30
20
10
0
*
*
0
5
10
15
Ada (μg/mL)
(b)
CD4
CD8
% CD25+ CD134+ T cells
100
80
60
40
20
0
0
1
2
5
Eta (μg/mL)
0
1
2
5
Eta (μg/mL)
CD4
CD8
% CD25+ CD134+ T cells
50
40
30
20
10
0
**
**
***
*
*
**
0
5
10
15
Ada (μg/mL)
0
5
10
15
Ada (μg/mL)
(c)
CD4
CD8
% Proliferating T cells
100
80
60
40
20
0
0
1
2
5
Eta (μg/mL)
0
1
2
5
Eta (μg/mL)
CD4
CD8
% Proliferating T cells
100
80
60
40
20
0
0
5
10
15
Ada (μg/mL)
0
5
10
15
Ada (μg/mL)

Supplementary Figure S2. In vitro effects of TNF antagonists on the functional capacity of T cells.

Whole blood and fluorescent dye-labelled PBMCs of 3 healthy donors (HD) were cultured with phytohemagglutinin (PHA, 5 μg/mL) and TNF antagonists were added at different concentrations (Eta at 1, 2, and 5 μg/mL, left panel; Ada at 5, 10, and 15 μg/mL, right panel). **(a)** The IFNγ production was assessed in supernatants after 18 hours of incubation. **(b)** The surface co-expression of CD25 and CD134 on $CD4^+$ and $CD8^+$ T cells was assessed after 44 hours of whole blood culture. **(c)** The proliferation of $CD4^+$ and $CD8^+$ T cells was evaluated after 5 days of incubation. The results are expressed as the mean ± SD of 3 independent experiments. Statistical analysis was performed using Student's *t* test.
